# Continuous Non-Invasive Arterial Pressure Assessment during Surgery to Improve Outcome

**DOI:** 10.3389/fmed.2017.00202

**Published:** 2017-11-17

**Authors:** Alena Stenglova, Jan Benes

**Affiliations:** ^1^Department of Anesthesiology and Intensive Care Medicine, Faculty of Medicine in Plzen, Charles University, Plzen, Czechia; ^2^Biomedical Centre, Faculty of Medicine in Plzen, Charles University, Plzen, Czechia

**Keywords:** blood pressure, non-invasive monitoring, volume clamp, vascular unloading, applanation tonometry, intraoperative hypotension, goal-directed hemodynamic therapy, postoperative outcomes

## Abstract

Blood pressure (BP) is one of the most important variables evaluated during almost every medical examination. Most national anesthesiology societies recommend BP monitoring at least once every 5 min in anesthetized subjects undergoing surgical procedures. In most cases, BP is monitored non-invasively using oscillometric cuffs. Although the risk of arterial cannulation is not very high, the invasive BP monitoring is usually indicated only in the case of high-risk patients or in complex surgical procedures. However, recent evidence points out that when using intermittent BP monitoring short periods of hypotension may be overlooked. In addition, large datasets have demonstrated that even short periods of low BP (or their cumulative duration) may have a detrimental impact on the development of postoperative outcome including increased risk of acute kidney or myocardial injury development. Recently marketed continuous non-invasive blood pressure monitoring tools may help us to recognize the BP fluctuation without the associated burden of arterial cannulation filling the gap between intermittent non-invasive cuff and continuous invasive arterial pressure. Among others, several novel devices based either on volume clamp/vascular unloading method or on applanation tonometry are nowadays available. Moreover, several near-future smart technologies may lead to better hypotension recognition or even prediction potentially improving our ability to maintain BP stability throughout the anesthesia or surgical procedure. In this review, novel or emerging technologies of non-invasive continuous blood pressure assessment and their potential to improve postoperative outcome are discussed.

## Introduction

Since the end of nineteenth century, when non-invasive monitoring using Riva-Rocci sphygmomanometer was improved and implemented into wide clinical praxis by Harvey Cushing, blood pressure (BP) became one of the three most important vital signs evaluated in the perioperative care. It is quite difficult to ascertain the contribution of BP monitoring to the improvement of postoperative outcome at that time, however, performing nowadays any anesthesia procedure without knowing patient’s BP is literally inconceivable. The American Society of Anesthesiologists (ASA) recommends in the Standards for basic anesthetic monitoring, that BP should be monitored in all anesthetized persons at least at 5-min intervals (1). The same recommendation (BP at least each 5 min) was incorporated into the World Health Organization’s “Guidelines for Safe surgery 2009” (2). Intermittent automated non-invasive oscillometric cuffs integrated into classic anesthesia monitors are mostly used for this purpose. This approach is convenient, safe, and reliable. However, motion artifacts, the need for adequate cuff size, and prolonged inflation/deflation times can pose significant drawbacks in routine care. The general perception of oscillometric non-invasive blood pressure (NIBP) accuracy has been also tempted (3). Until recently, more reliable and in particular continuous BP monitoring has been possible only using arterial catheterization (A-line) and direct pressure measurement. The arterial cannulas are usually well tolerated and pose only limited risk to the patient (4), but still this technique is usually limited to the high-risk cases only. However, even among high-risk surgical patients in about 50% the NIBP is used (5).

Using the intermittent cuff, NIBP monitoring may leave BP fluctuations undetected or may lead to late recognition and delayed correction (6, 7). Several recent large scale observational studies have demonstrated, that not only the “intensity” (depth of hypotension) but also the “dose” (cumulative time spent in hypotension) are associated with severe postoperative complications [myocardial infarction, stroke, or acute kidney injury (AKI)] (8–11). Recently, several monitors enabling for continuous non-invasive blood pressure (CNBP) monitoring have been marketed. These new technologies combine the advantages of both non-invasive cuffs and arterial catheters. They offer reliable real-time estimation of actual BP and display pressure curve making advanced analyses possible (i.e., calculation of pulse pressure variation, maximal pressure change, or hemodynamic variables using pulse contour/power analysis). Further use of smart technologies and software prompts enables not only fast recognition but even prediction of further BP course decreasing the risk of hypotension-associated complications. In this review, we discuss several novel aspects of up-to-date BP monitoring and their possible impact on patients’ outcome.

## Intraoperative Hypotension (IOH) and Perioperative Outcome

In this literature, we may find numerous definitions of IOH. Bijker et al. have identified 140 different definitions in 130 studies (12) ranging from systolic blood pressure (SBP) below 100 mmHg to a complex definition based on absolute SBP and mean arterial pressure (MAP) values and their relative decrease to baseline. Naturally, the incidence of IOH varied significantly (from 5 to 99%). The authors of that study suggested a dynamic approach to the IOH, rather than arbitrarily chosen thresholds (12). As an example of answering individual needs of pressure targets, the SEPSISPAM study may serve to show the profit of higher pressure in the critically ill with chronic hypertension (13). Several other authors have studied the issue of IOH and increased risk of organ complications.

Salmasi et al. (9) have demonstrated on a large database (57,315 non-cardiac surgery patients) that risk of acute kidney injury (AKI) and myocardial injury (MI) starts to increase when intraoperative BP declines below 65 mmHg or more than 20% from baseline (defined as an average of all MAP readings over 6 months prior hospitalization). The risk further increased with profound hypotension. Besides, the effect was “time-dose” dependent. Similar pattern of AKI and MI risk increase, but with the lower threshold (MAP of 55 mmHg) was observed by Walsh et al. (8) in another large retrospective single-center database cohort (33,330 non-cardiac surgery patients). These findings were further supported in a prospective way by Sun et al. (11), who found a strong association between AKI development and MAP < 60 mmHg lasting more than 20 min or MAP < 55 mmHg more than 10 min. None of these studies have performed separate analysis in patients with chronic hypertension [though they created 48% in Sun et al. (11) and 49% in Salmasi et al. (9)], albeit the higher risk was observed in these patients.

The association between low intraoperative pressures and increased risk of vascular brain injury (namely stroke) and increased mortality was stressed by the results of the POISE trial (14). The extended release metoprolol administration was protective against MI in elective non-cardiac surgery patients, but it led to increase in stroke incidence and death in patients with a history of cardiac, peripheral artery disease, or stroke. IOH associated with metoprolol administration was deemed to be the culprit of this unfavorable outcome of this prospective randomized trial. In another large population retrospective (48,241 non-cardiac and non-neurosurgical patients), Bijker et al. (10) supported this association. Each minute of IOH defined as a MAP drop of more than 30% from baseline increased the odds ratio of postoperative stroke within 10 days after surgery by 1.013 (95% confidence interval 1.000–1.025).

Intraoperative hypotension and higher occurrence of organ complications may be also linked to increased postoperative mortality in non-cardiac surgery patients as demonstrated by Mascha et al. (15). Naturally, the pressure thresholds were much lower to induce fatal complications. Time-weighted average (TWA) of MAP equal to 50 mmHg increased the 30-day mortality more than three times compared to 80 mmHg. Interestingly, short-time variability of BP had much lower effect than long-term trends. In the retrospective analysis of 46,496 procedures performed on 30,650 patients in six American Veteran hospitals by Monk et al. (16), IOH, but not hypertension, was coupled with increased 30-day mortality after major non-cardiac surgery. Thresholds found in this were basically similar to Mascha et al. (15): absolute SAP < 67–70 mmHg or MAP < 49 mmHg for more than 5 min and relative MAP drop more than 50% of baseline for 5 min.

Based on all these large population samples, the risks of IOH are undeniable, especially in non-cardiac surgery patients. Moreover, the inconsistency of IOH thresholds leading to different complications may be attributed to different organ needs and population under study. The threshold of AKI and MI increased risk of MAP below 60–65 mmHg corresponds with the lower inflection point of renal and myocardial autoregulation curves. Because the brain autoregulation’s plateau starts at lower MAPs, the threshold observed is lower (drop of more than 30% of chronic MAP). Finally, the burden of global hypoperfusion has to be much higher to induce life-threatening situation—i.e., TWA MAP 50 mmHg corresponds with profound hypotension throughout the procedure as well as SAP lower than 70 mmHg or MAP drop of 50%. Therefore, nowadays, the question should not state: “Is IOH dangerous?” but “How the IOH could be prevented…” Several hints may be already found in the literature. First the “Triple low study” (17) and its followers (18, 19) have demonstrated, that unnecessarily deep anesthesia in frailty individuals may significantly contribute to the risk of IOH with its consequences. More recently, the retrospective analysis from Germany (20) pinpointed that not every IOH is the same: the IOH within 20–30 min after induction (post-induction hypotension) has slightly other background than IOH occurring later on. Low pre-induction SAP, older age, and emergency surgery contributes to both types of IOH, but the use of supplementary epidural or spinal anesthesia, male sex, and the American Society of Anesthesiologists physical status grade 4 was associated with hypotension occurring later on during the procedure. Another possibility is to use continuous BP monitoring which may help to identify hypotensive periods more swiftly and hence decrease the time dose (7).

## Contemporary Possibilities of Continuous NIBP Assessment

**Figure 1:**
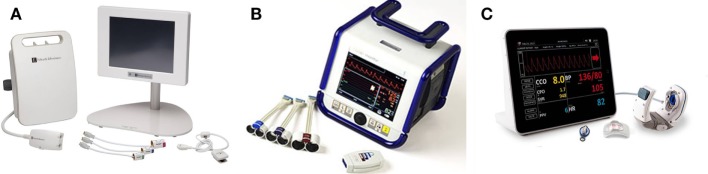
Currently available continuous non-invasive blood pressure monitors: **(A)** ClearSight with EV 1000 monitoring platform, **(B)** CNAP HD device, **(C)** T-line 400 device. Device photographs for publication’s purpose were provided with permission to re-use by the manufacturers or distributors.

Since the second half of twentieth century, several technologies of continuous NIBP assessment have been available. Unlike the occlusive technique used in standard pressure cuffs (both Riva-Rocci–Korotkoff and oscillometric methods), these techniques are non-occlusive based on pressure transduction over the vessel wall under dedicated conditions. Important base for this research was Etienne-Jules Marey’s development of former Vierordt’s sphygmograph into portable form in 1860. In his later works, Marey described the relationship between the amplitude of pulse and pressure imposed on the vessel wall from the outside: i.e., the largest oscillations are observed in the moment of zero transmural pressures. Besides, the contemporary technologies of continuous NIBP monitoring (Figure 1) are based on two major principles: volume clamp and applanation tonometry. Numerous validation studies were performed under divergent conditions; their results are so far not entirely satisfactory as demonstrated by large meta-analysis by Kim et al. (21). On the other hand, there is currently no widely accepted standard or methodology how to evaluate the accuracy of such new devices and the Association for the Advancement of Medical Instrumentation (AAMI) standard (22) does not seem to be the best option (23).

### Volume Clamp Method

The Czech physiologist Jan Peňáz first described the volume clamp method in 1973. In this semiocclusive technique, the volume of finger arteries is assessed using infrared photo-plethysmography. Next, using fast reacting inflatable pressure cuff, the volume of blood is held constant. The pressure which is needed to maintain a constant blood volume is proportional to the BP. To obtain real BP values (not only a proportional estimate), the zero transmural pressure needs to be obtained. Under zero transmural pressures (the so-called vascular unloading), the pressure outside (i.e., in the finger cuff) and inside the vessel are equal hence enabling the reconstruction of BP curve and assessment of numerical values. Based on the Marey’s experiments, the zero transmural pressure is accompanied by the maximal amplitude of pulse oscillations. However, the vascular tone may change in time making the vascular unloading far from being constant. In the 1995, Karel Wesseling developed the Physiocal™ algorithm for automatic vascular unloading set point assessment that leads to gross improvement in the device accuracy. The device enabling non-invasive finger cuff was later marketed under the name Finapress/Portapress. Nowadays, different methods of vascular unloading are used by divergent devices. Because the pressure tracing monitored using this technique corresponds with the pressure inside finger arteries, a further mathematical processing is needed to reconstruct either radial or better brachial pressure curve or values.

A higher than venous pressure inside the finger cuff leads to venous congestion distally to the probe. This so-called blue finger syndrome is mostly regarded as unpleasant or disturbing. In any case, it may limit the length of the monitoring in conscious subjects. Under several non-frequent conditions—as for instance Raynaud’s syndrome—this method of pressure monitoring is better to be avoided. The accuracy of the monitoring may be significantly affected in patients with finger edema or low perfusion due to blood redistribution (low cardiac output), chronic vascular disease, or peripheral vasoconstriction (hypothermia, shock states).

#### ClearSight (Former Nexfin)

ClearSight technology marketed by Edwards Lifesciences Inc. (Irvine, CA, USA) is a direct successor of former Finapress and Nexfin (BMEYE B.V., Amsterdam, The Netherlands) devices encompassing the Physiocal™ vascular unloading algorithm. A disposable single-use cuff is placed around the second phalanx of finger (usually index, but middle or ring finger use is also possible) connected to a band held pressure controller. The pressure inside the finger cuff is determined by the photo-plethysmographic sensors inside the cuff at a rate of 1,000 Hz. Within a time span of 5–70 beats the set point is reassessed using Physiocal™ algorithm. Mathematical inversed transfer function reconstructs the brachial BP curve out of the finger tracing and heart reference system is available to eliminate inaccuracies induced by hand vertical movements. Using the pulse contour analysis (adapted Modelflow method), advanced hemodynamic variables are calculated from the reconstructed pressure curve. The results of validation studies concerning BP and cardiac output accuracy performed using Nexfin device are also applied to the ClearSight, because this technology is a direct successor of the former one.

#### CNAP

The CNAP device (CNSystems, Graz, Austria) is second currently available device based on the Peňáz’s principle. In contrast to the ClearSight, the finger probes of CNAP are more robust and durable. Two neighbor fingers (either index and middle or middle and ring finger) are inserted into a double lumen plastic tunnel encompassing the inflatable finger cuffs. This setting enables periodical finger switch and to avoid the prolonged venous congestion of the acral part. A system of interlocking control loops (VERIFIY algorithm) is used for optimal vascular unloading. Upper arm oscillometric cuff calibration (or any other external input) is necessary for brachial pressure reconstruction. According to the manufacturer, such calibration should be performed in 15- to 30-min window; however, frequent recalibrations (each 5 min) are probably more appropriate to maintain adequate accuracy (24). However, the inaccuracy of the oscillometric cuff pressure reading mentioned previously (3) may concomitantly affect accuracy of CNAP monitoring, especially in high and low BP range too. The most recent device version (CNAP HD) provides calculation of hemodynamic variables.

### T-Line

The T-line (Tensys Medical Inc., San Diego, CA, USA) is the last commercially (and globally) available option of continuous NIBP monitor. Unlike previous ones, T-line is based on radial arterial wall applanation based on the Pressman and Newgard device described in the 1963 (25). A pressure transducer is placed over an artery supported by a bony structure hence enabling its compression (applanation). For T-line, a reusable bracelet with singe use interface is placed over the wrist, enabling a close contact between the sensor and radial artery. Based on the third Newton’s law, the pressure inside is directly proportional to the force which induces flattening of a ball surface and indirectly proportional to the area of contact. Creating a zero transmural pressure leads to obtaining maximal pulsations and hence accurate MAP assessment. A mathematical correction for elastic tissues lying between the artery and sensor is needed [the detailed technology is described in the original article by Pressman (25) and in the excellent review by Matthys et al. (26)]. An important drawback to the technology is the extreme sensitivity of the sensor position; therefore, two servo motors automatically and continuously reassess the sensor position. Similar to previous devices, the reconstructed arterial wave enables calculation of different advanced hemodynamic variables, including cardiac output (27). Several validation studies exist for the different T-line generations, mostly with accuracy comparable to volume clamp devices as demonstrated in the meta-analysis by Kim et al. (21).

## Non-Invasive Pressure Assessment to Improve Outcome

**Figure 2:**
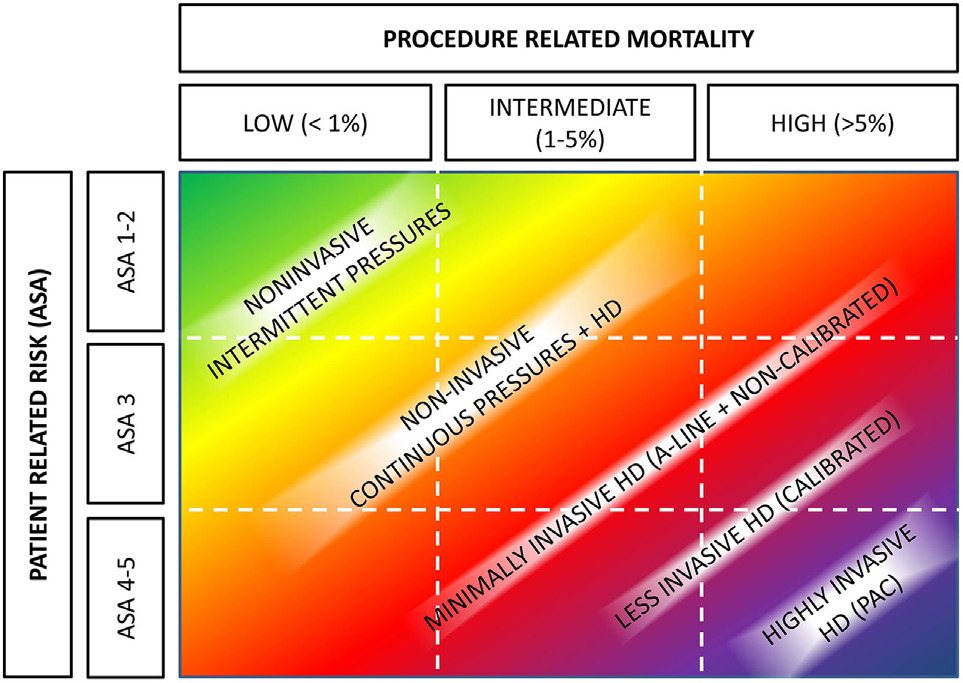
Hemodynamic monitoring based on patients’ and operative risks. Abbreviations: A-line, arterial cannulation; ASA, American Society of Anesthesiologists physical status; HD, hemodynamic monitoring. Authors’ own design based on Kirov et al. (28).

Basically, the described CNBP monitoring tools may help to improve perioperative care in two ways. First to replace contemporary invasive means and second to improve monitoring in patients who were deemed too good to have such invasive BP assessment. In 2012, Kirov et al. (28) have proposed a two-dimensional decision table for intraoperative monitoring. Given current possibilities, this table may be adapted into current form (Figure 2).

The first option, decreasing monitoring associated burden in patients currently monitored using invasive arterial pressure, seems to be far less important in the clinical routine. First, the risks associated with arterial cannulation, especially radial, are not negligible (4), but rather small and easily outweighed by the risks of the procedure. Second, the A-line is inserted not only for BP monitoring but also to facilitate blood sampling and gas analysis, things not possible with CNBP. And finally, the CNBP readings would have to be fully reliable under all conditions. Adherence to the AAMI standards would not help us in this issue (23). The validations of current CNBP devices have been performed using the old Bland–Altman methodology, but possibly we should go further into more elaborate analyses using error grams (29), four-quadrant, and polar plots (30) as described by Critchley. At any circumstances, the reliability of current CNBP seems not to reach this (21).

From this perspective, the second option (increasing the spectrum of monitoring in “good patients”) might be far more clinically relevant. Because of the intermittent nature of NIBP, BP fluctuations may be missed. In 2012, Chen et al. (6) have demonstrated that as monitored by Nexfin device in average 7 ± 1 min of hypotension and 7 ± 2 min of hypertension per 1 h of general and orthopedic surgery time were missed when NIBP with 5 min period was used. Later on that year, Ilies et al. (31) used CNAP device during Cesarean section under general anesthesia and observed similar results: CNAP was able to identify hypotensive periods (SAP < 100 mmHg) in 91% of parturient (as compared to 55% by NIBP each 3 min) with prolonged duration. It is important to note that the umbilical venous pH was significantly more deranged in these newborns whose mothers were identified to be hypotensive by CNAP. In both these trials, CNBP devices were used to monitor, but not to intervene, the BP fluctuations. In another study, Benes et al. (7) have compared CNAP device to NIBP (at least each 5 min) in a randomized fashion to intervene BP fluctuations in patients undergoing thyroid gland surgery in half-sitting (beach chair) position. The results have clearly demonstrated that using continuous monitoring time spent in hypotension (20% decrease from preoperative values) may be significantly shortened (12 [4–20] vs. 27 [16–34] min), although not eliminated. Finally, recent randomized trial by a German group has demonstrated that use of CNBP even without any dedicated protocol led to higher BP stability and fewer hypotensive events (32). However, none of these trials has demonstrated any clinically relevant benefit in CNBP monitored patients. The only data demonstrating that maintaining BP in range ±10% of patient’s resting systolic BP in major surgery has impact on postoperative organ dysfunction by day 30 as compared to standard care come from recently published INPRESS trial (33). Patients at risk of renal dysfunction were studied and radial arterial cannulation was used to monitor continuous BP in this trial. Hence, the real clinically relevant impact of decreased IOH occurrence based on CNBP monitoring on postoperative outcome (organ dysfunctions, etc.) in intermediate risk patients is still speculative and opens a wide arena of possibilities for future research.

However, decreasing the risks of IOH is not the only possibility how CNBP devices may impact on rate of postoperative complications. Given the reconstruction of arterial curve, a beat-to-beat analysis of hemodynamic variables and/or their induced fluctuations are inevitably part of the displayed information. Variation in pulse pressure (PPV) induce by mechanical ventilation has been shown to be an excellent predictor of fluid responsiveness (34). The use of invasive PPV (or its surrogates) for goal-directed fluid therapy (GDFT) has been associated with improved outcomes in high-risk surgical patients (35). Moreover, the PPV assessed using CNBP devices seems to be as accurate as the invasively obtained one (36–38). Based on these findings, it seems rational that GDFT principles may be transposed to lower risk patients’ groups. So far, two studies have been published proving such concept, but multiple others are ongoing (for example, NCT02950649, NCT02135146, NCT02382185, NCT02479321, NCT02343601, and NCT03189550). In our institution, we have started to implement CNAP device for intraoperative monitoring of patients undergoing total hip or knee replacement (39). A before-and-after evaluation revealed significant decrease in transfusion needs and resulting number of infectious and organ complication in the GDFT group managed using PPV as compared to historical control (39). More recently, Broch et al. (40) have published results of their GDFT study using Nexfin device. On a small sample size, the authors were able to demonstrate the feasibility of the concept of non-invasive GDFT, naturally because of small numbers included, they have failed to demonstrate improvement in patients’ outcome (40).

At any case, use of CNBP devices for intraoperative hemodynamic care seems to offer a large field of small improvements in patients’ care and may be deemed as a natural part of current and future Enhanced Recovery programs. However, it should be noted that at current state large outcome data (i.e., mortality or morbidity benefit) as well as cost-effectiveness studies are missing. This coupled with price of the equipment and/or disposables create a not negligible impediment in routine use. At the end of the day, BP and flow are only global hemodynamic indicators and possess only limited information about end-organs perfusion and tissue metabolic well-being. Future clinical research should therefore try to couple these macrohemodynamic indices with monitoring of organ perfusion and assess impact of both these factors on patients’ postoperative outcome.

## Emerging and Future Concepts

Because the ability to assess the patients’ hemodynamic status is so appealing for the domain of anesthesiology, perioperative and intensive care multiple further technologies are in the pipeline of development. Practical applications based on pulse transit time (41, 42) and pulse decomposition analysis (43, 44) are currently available even though their validations for given field is still insufficient and probably multiple improvements in mathematical models used will be necessary prior clinical routine use. Besides, several patents are placed on use of superficially placed optical (patent US 20050228299A1), piezoelectric (45), or mechanical (surface acoustic wave—patent US 20110208066A1) continuous non-invasive pressure sensors. As pointed recently in futuristic views of hemodynamic monitoring in the 2050 will be “NEWS”—Non-invasive, Easy to use, Wireless and wearable, and first of all Smart (46).

Such Smart software development may significantly alter the way patients will be monitored in the future. Over the past decade, we have significantly improved the way how we analyze the arterial pressure curve, but still the modality is not fully exploited. Assessing dynamic arterial elastance to predict pressure response on fluid administration is still in its basics (47), but may play important part in future decision-making how to treat hypotensive periods in the future. Use of closed-loops systems to deliver fluids (48) or vasopressors (49, 50) is now limited in their clinical applicability, but when combined with neuronal networks able to recognize the source of hemodynamic instability may open the door for their routine use. A combination of more information sources together (i.e., pulse transit time, finger volume clamp, and surface sensor) may further improve the way we perform hemodynamic monitoring. For instance, taking together more vital signs (like the Vital Sign Index by Visensia™ monitor, OBS Medical, IN-USA) may help to predict cardiac instability (51) or assessing the heart rate variability from electrocardiography may be useful in predicting hypotension (52). Another example may be the recently approved Hypotension Probability Indicator by Edwards Lifesciences Inc. which should be able to predict hypotension based on analysis of multiple domains including arterial pressure curve complexity, heart rate variability, and others by proprietary algorithm combined with machine learning. Merging non-invasive hemodynamic (not only pressure) sensors with automated signal analysis may promote current trend of expanding postoperative intensive care into the standard wards or even home without decreasing patients safety (53).

## Conclusion

Blood pressure monitoring is a vital part of perioperative care. Current technologies (although not perfect) enable much wider application of continuous monitoring hopefully leading to decrease in undesired BP fluctuations and hypotensive periods. Sophisticated analyses of arterial pressure curve make possible to monitor not only BP but also blood flow (and its variations). These new monitoring tools available today may significantly influence perioperative care especially in intermediate risk patients. However, to which extent the macrohemodynamic parameters improvement impact postoperative outcome in this patient population has to be determined in forthcoming studies. Future developments in this field coupled with smart technologies and in conjunction with other possibilities to assess end-organ perfusion may further improve patient care.

## Author Contributions

AS and JB both wrote the manuscript, performed the literature search needed, and approved the final form of the text.

## Conflict of Interest Statement

JB is in a long-term relationship with Edwards Lifesciences Inc. and CNSystems Medizintechnik AG (including speaker and travel fees, advisory board membership and institutional publication grants and device lending). AS has no conflicts to declare.

## Abbreviations

AAMI, Association for the Advancement of Medical Instrumentation; AKI, acute kidney injury; A-line, arterial cannulation; ASA, American Society of Anesthesiologists; BP, blood pressure; CNBP, continuous non-invasive blood pressure; GDFT, goal-directed fluid therapy; HD, hemodynamic monitoring; IOH, intraoperative hypotension; MAP, mean arterial pressure; MI, myocardial injury; NIBP, non-invasive blood pressure; PPV, pulse pressure variation; SBP, systolic blood pressure; TWA, time-weighted average.
